# 

**Published:** 1914-05-30

**Authors:** 


### Our Next Competition.

**Our readers are referred especially to page iii of the present issue for particulars of this competition, where they will find the prizes offered, and the conditions, which cannot fail to be of practical interest.**

### Medical Appointments.

The following appointments have been made at the Manchester Children's Hospital: Dr. Thos. Hayhurst, M.B., Ch.B. Edin., senior resident medical officer at the hospital at Pendlebury, and Dr. George Jackson, M.B., Ch.B., junior resident medical officer. Dr. A. H. Miller, M.A. Camb., M.R.C.P. Lond., has been appointed pathologist.

Dr. C. G. H. Campbell and Dr. G. Davidson have been appointed by the Metropolitan Asylums Board junior assistant medical officers in the infectious hospitals service. Dr. Campbell has the degrees of B.A., M.R.C.S., L.R.C.P. (Cantab.), and first two sections of M.B., B.C. (Cantab.). He has been assistant house physician and house physician (nine months) and assistant house surgeon and house surgeon (nine months) at the Westminster Hospital, and also clinical assistant at the Liverpool Children's Infirmary, and has acted since April 16 as temporary assistant medical officer at the Downs Sanatorium. Dr. Davidson has the degrees of M.B., Ch.B., D.P.H. (Aber.). He has been assistant house surgeon at the Huddersfield Infirmary for eight months, junior and senior resident medical officer at the Paddington Infirmary (two years), and has acted for six months as ship's surgeon.

RATES OF SUBSCRIPTION: Twelve months, 6/6; Six months, 4/-; Three months, 2/-, post free to any address in the United Kingdom. Abroad, Twelve months, 8/8; Six months, 5/-; Three months, 2/6, post free.—Address The Subscription Dept., "THE HOSPITAL," The Hospital Building, 28/29 Southampton Street Strand, London, W.C.

